# HDL May Improve Ocular Tear Film Stability in Patients with Gastric Bypass: A Pilot Study

**DOI:** 10.3390/diagnostics15202581

**Published:** 2025-10-13

**Authors:** Anabel Sanchez-Sanchez, Ma Guadalupe Leon-Verdin, Alberto Hidalgo-Bravo, Claudia Martinez-Cordero

**Affiliations:** 1 Instituto Nacional de Astrofísica Óptica y Electrónica, Tonantzintla, Puebla 72840, Mexico; optiani@hotmail.com; 2 Instituto de Salud Pública del Estado de Guanajuato, Guanajuato 36000, Mexico; lupitalv2001@gmail.com; 3 Departamento de Medicina Genómica, Instituto Nacional de Rehabilitación, Ciudad de México 14389, Mexico; dr_genetica@yahoo.com; 4 Hospital Regional de Alta Especialidad del Bajío, Servicios de Salud del Instituto Mexicano del Seguro Social para el Bienestar (IMSS-BIENESTAR), León 37544, Mexico

**Keywords:** ocular tear film, gastric bypass, cholesterol, triglycerides, HDL

## Abstract

Most people with obesity who have undergone gastric bypass surgery have dyslipidemia. Because tear film layers play a major role in the pathogenesis of evaporative dry eye, some studies suggest that dry eye syndrome (DES) and dyslipidemia could cooperate in the ocular system. This study aimed to investigate whether tear film conditions are correlated with blood lipid levels. We calculated a sample of 29 patients in this study. We measured the characteristics of the tear film via the Schirmer test and tear break-up time (BUT) test; three measurements were made, and the average value was subsequently recorded. High-density lipoprotein (HDL) correlated positively with BUT (*p* < 0.05), but cholesterol and triglycerides correlated negatively with Schirmer (*p* < 0.05 and *p* < 0.001, respectively). Our findings suggest that HDL levels significantly influence ocular tear film stability and that triglycerides and cholesterol influence the aqueous component of the tear film, which is conducive to the hypothesis that postgastric bypass surgery tear deficiencies could be mainly of evaporative origin and not watery. Bariatric patients may have a high likelihood of suffering dry eye by modifications in the lipid tear layer; however, the development of DES in bariatric patients remains unclear. Thus, high levels of cholesterol and triglycerides could be associated with aqueous-type dry eye (main gland production), and low HDL levels could be associated with evaporative-type dry eye (meibomian gland production).

## 1. Introduction

Most patients with obesity who have undergone gastric bypass surgery present tear film disorders. The tear film, which is formed by blinking and covers the corneal surface with a layer of approximately 3–10 microns, is completely necessary for the anterior segment of the eyeball. This film creates a smooth, refractive surface on the cornea, the first optical element of the eye, allowing a sharp image to be obtained. One additional reason is that it plays a crucial role in protecting the corneal surface, providing a homogeneous and smooth surface on the cornea to work in an optically correct way and ensuring that something is not harmed, injured, or damaged, with antimicrobial action and nutritional contributions. This thin layer is composed, in turn, of three thinner sublayers: one of mucin, another of aqueous (mostly made up of water), and the last of lipids or fats. Each of these layers originates from different glands located in the temporal fossa and in the eyelids, and the work of blinking combines these three components, thus forming the tear film. The formation and stability of the tear film depend on the composition and natural proprieties of the tear in both healthy and diseased eyes [1]. The meibomian glands are the main producers of the lipid layer, which may be responsible for the stability of the tear film by reducing the process of the liquid layer changing into a gas in the open eye [2].

Three different processes are involved in the anatomical structures of the lacrimal system: production, distribution, and excretion of the tear film. The three layers involved in the process of making the tear film are lipids, aqueous liquids, and mucous. Gayton defined tears as interactive gels of hydrated mucin with lipids and protein associated and distributed [3]. Tear film plays a crucial role in the health and homeostasis of the anterior segment of the eyeball. Additionally, the tear film contributes optically by forming a regular layer on the cornea while also lubricating and defending the eye from microbes and preserving the health of the cornea and conjunctiva owing to its enzyme content and pH level.

In a clinical case, one woman presented severe opacity in the cornea and low HDL in the blood [4]; on the other hand, an epidemiological study revealed that a higher level of HDL favors the presence of drusen in age-related macular degeneration [5]. With particular attention to the ocular surface, it has been suggested that cholesterol levels may affect the homeostasis and pathophysiology of lipids on the ocular surface [6]. Specifically, low HDL levels could be associated with an increased risk of dry eye. Although one study reported that women with hypercholesterolemia have a greater prevalence of tear deficiencies [7], no studies have reported tear film deficiencies in persons with gastric bypass surgery under normal blood lipid conditions.

The Schirmer test and BUT test estimate different properties of the tear film: the Schirmer test assesses the quantity of the tear aqueous element, which is produced mainly by the lacrimal gland, whereas the BUT test evaluates the tear lipid element produced mainly by the meibomian glands that help to stabilize the tear film [2,8]. The importance of evaluating these aspects of the tear film is that water and lipid deficiencies have been classified as the main causes of dry eye [9]. Meibomian glands principally produce a lipid layer that prevents evaporation and helps to stabilize the tear film. The aqueous layer is produced to retain moisture on the ocular surface, and the main lacrimal gland is responsible for producing this layer. Some studies have associated dyslipidemia and dry eye, including dyslipidemia and dysfunction of the meibomian glands [7,10]; in particular, the effects of high-density lipoprotein (HDL) on the eyeball have not been studied. Our research group has investigated several aspects of eye health in patients with severe past obesity who were subsequently subjected to gastric bypass surgery [11,12]. This exploratory, cross-sectional, observational correlational study in a Mexican hospital focused on tear film production and stability in a group of people who underwent bariatric surgery and had a normal lipid level. In addition, gastric bypass surgery has been reported to improve all the elements of the lipid profile in those who undergo it [13]. Therefore, the aim of this study was to investigate whether tear film conditions are correlated with blood lipid levels in people with gastric bypass inside normal values. Additionally, a comparison with other studies performed on people without gastric bypass surgery with normal lipid levels was performed.

## 2. Materials and Methods

This exploratory protocol was approved by an ethical committee (CI/HRAEB/044/2020), and the patients participated after signing a written consent form. For this pilot study, we calculated a sample of 29 patients with 95% confidence and a significance of 10%; in a public hospital, we invited all patients who underwent gastric bypass surgery to participate. As a result, 30 female adults who underwent surgery were included. The selection criteria were male and female patients with a postsurgical duration of at least two years, cholesterol ≤ 200 mg/dL, triglycerides ≤ 150 mg/dL, HDL ˃ 40 mg/dL, and glucose ≤ 110. The exclusion criterion was patients with associated systemic disease, specifically diabetes or metabolic syndrome, due to the drug’s effects on the lacrimal glands and/or corneal diseases.

**Figure 1:**
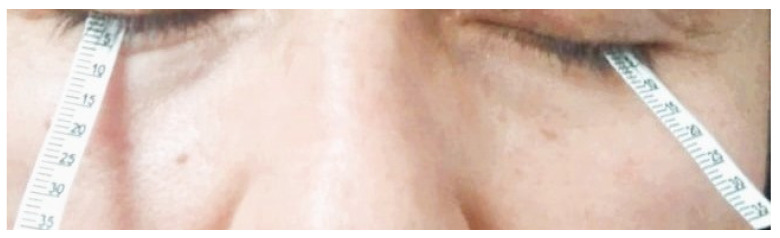
Schirmer’s test. Describe the placement of the Schirmer strips on the outer third of the eyelid.

Before the tear film tests, we collected a fasting blood sample to determine the blood chemistry and lipid profile. We assessed the tear film with the Schirmer test and the BUT [14]. For the Schirmer test, without anesthesia, we gently placed sterile tear flow test strips 5 × 35 mm (Tear Flo, HUB Pharmaceuticals, LLC., Scottsdale, AZ, USA) in the outer third of the lower eyelid in each eye while avoiding contact with the cornea. We started the chronometer when the patients closed their eyes, and ocular movements were minimally controlled during this time; then, we removed the bands after five min, and we measured the strip wetting in millimeters (Figure 1).

**Figure 2:**
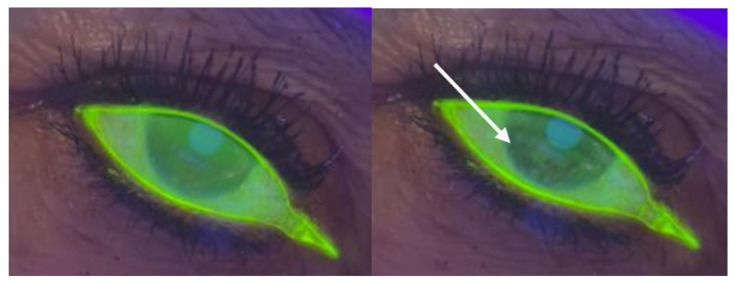
The BUT test (**on the left**), the beginning of the test, with a regular distribution of fluorescein (**on the right**), the white arrow indicates the appearance of black spots and rupture of the tear film stability can be observed.

For the BUT test, we used a Burton lamp and sterile fluorescein strips (Bio-Glo-HUB Pharmaceuticals, LLC., Scottsdale, AZ, USA). Once the fluorescent substance was distributed and homogenized on the anterior surface, the patient was asked to close and open the eyes three times, and the chronometer was activated at the same time. Chronometry was stopped when we observed the black spots or cracks that appeared in the tear film with fluorescein via a Burton lamp. The measurements were made three times, and the average time was subsequently recorded (Figure 2).

To observe whether the trends in the results obtained in our bariatric surgery group were similar to those in the nonbariatric surgery group, the results of a similar study reported by Erdur et al. (2017) were considered a control group [15]. We performed the data analyses via SPSS 27 software. We used the Spearman Rho value for correlations within the bariatric surgery group. Welch’s *t*-tests were also used to compare HDL, triglyceride, Schirmer, and BUT levels between the gastric bypass surgery group and the Erdur’s control group. The data are routinely presented as the means and SDs.

## 3. Results

The participants’ general data are described in Table 1. Blood results were not obtained for the 30 patients who responded to the call, so the number of patients included in each analysis is shown in Table 2.

For statistical analysis, Table 2 shows the values obtained from the tear film scores and lipid profiles of the samples in this study and the results described by Erdur et al. for the control group [15]. The control group included patients without gastric bypass surgery and with normal lipid levels. Welch’s t-test results for comparing groups are presented in Table 2. Although there was a difference of more than five units for HDL and one unit for triglycerides, these differences were not statistically significant (*p*-value > 0.05). These findings indicate that blood HDL and triglyceride levels were not significantly different between our group and Erdur’s group. For the tear film score, the mean difference for Schirmer was only 0.2 units, whereas the difference for the BUT was greater than 13 units. In these comparisons, only the BUT was significantly different between the groups. Glucose levels were not compared because, for both groups, they were within normal values and were not lipids.

**Figure 3:**
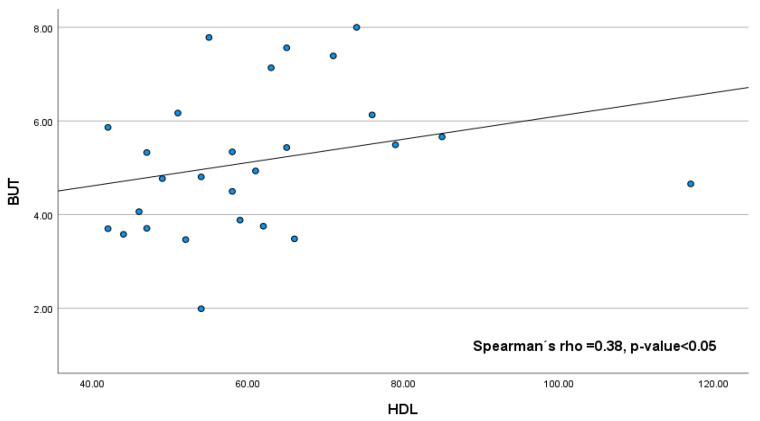
HDL vs. BUT. This study revealed a positive correlation between break-up time and HDL level.

**Figure 4:**
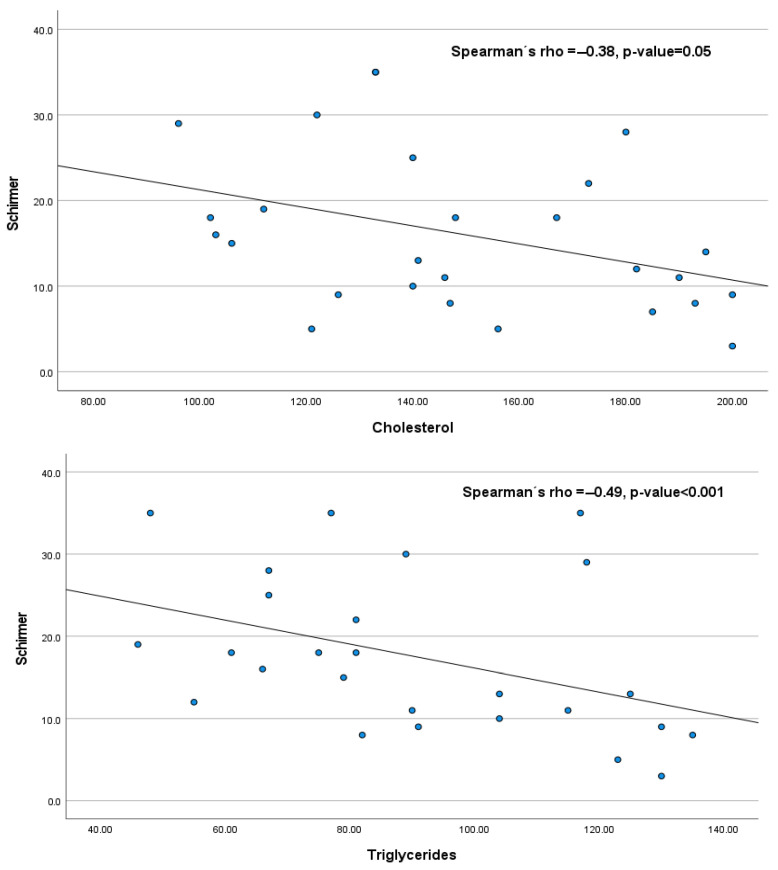
Up Schirmer vs. triglycerides. Down Schirmer vs. cholesterol. Both have negative correlations.

Figure 3 and Figure 4 show the statistically significant correlations between the tear film test scores and lipid levels in our bariatric group: high-density lipoprotein (HDL) correlated positively with BUT (Spearman’s rho = 0.38, *p*-value < 0.05); however, there was a negative correlation between cholesterol and Schirmer (Spearman’s rho = −0.38, *p*-value < 0.05) and triglycerides and Schirmer (Spearman’s rho = −0.49, *p*-value < 0.001). The correlations between the tear film test scores and lipid levels were not statistically significant: cholesterol vs. BUT, Spearman’s rho = 0.21; triglycerides vs. BUT, Spearman’s rho = −0.04; and HDL vs. Schirmer, Spearman’s rho = −0.02.

## 4. Discussion

Our findings suggest that HDL levels significantly influence ocular tear film stability and that triglycerides and cholesterol influence the aqueous component of the tear film. First, Li Y et al. suggested that low HDL levels are associated with an increased risk of dry eye [16], and this finding coincides with our results that the higher the HDL value is, the higher the BUT value is, thus favoring the stability of the tear film. Serrano Morales et al. reported that HDL values greater than 40 mg/dL have a protective effect against dry eye [10], and these findings are consistent with our results related to the positive correlation between BUT and HDL. In contrast, Chun YH et al. reported that high HDL could be a risk factor for dry eye disease (DED), as their results were based on the application of a questionnaire without any tear test [7], so it is not possible to contrast the studies. Given that the values of glucose, cholesterol, triglycerides, and HDL in our study correspond to values within the normal range and that, on the one hand, the BUT values are positively correlated with HDL, it can be inferred that the effect of HDL is favorable for increasing the value of the BUT. In this sense, HDL, in addition to protecting the heart, could have an important contribution to the stability of the tear film. Additionally, the low BUT values in Table 2 possibly indicate that meibomian gland functions could be affected after bariatric surgery.

Second, the Schirmer values are negatively related to cholesterol and triglyceride levels. A study carried out by Serrano Morales et al. revealed that individuals who described a greater presence of DED symptoms presented elevated cholesterol and triglyceride levels [7]. In this same study, people with high levels of cholesterol, LDL and triglycerides presented greater loss of meibomian glands in the lower eyelid. Tzu-Hao et al. reported an association between dyslipidemia and DED [17]. Chun et al. reported that, with respect to the lipid profile, only elevated cholesterol is related to DED in women [7]. Other studies have reported a greater presence of DED symptoms with elevated cholesterol levels [7] in women. Thus, the results of the studies described above are consistent with those obtained in this study from the point of view that at higher levels of cholesterol and triglycerides, the Schirmer values decrease. These findings suggest that cholesterol and triglyceride levels could influence the production of the aqueous layer of the tear film, which is considered one of the etiologies of DED. In contrast, a study carried out by Serrano Morales et al. described a positive relationship between cholesterol and the height of the tear meniscus [7] and reported that tear menisci constitute between 75% and 90% of the total volume of tear fluid. The tear meniscus measurement is considered an estimate of the aqueous layer of the tear film [18]. This is a measure that helps in the classification of dry eye. Importantly, there is still a long way to go in relation to cholesterol and triglyceride levels and their effects on the production of the aqueous components of the tear film. Another study by Kennet et al. reported similar results. No relationship between symptoms of hypercholesterolemia and DED [19] has been reported.

When we compared our results from patients who underwent gastric bypass surgery with the data reported by Erdur et al. (2017), both patients with and without gastric bypass surgery presented normal lipid levels in both groups and no significant differences in HDL or triglyceride levels [15]. For cholesterol, the comparison was not possible since this value was not reported by them in their study. In relation to the tear tests, no significant differences were observed in Schirmer, and the only test that showed significant differences was the BUT between the groups compared. It is not possible to compare other details, since the objectives described by Erdur were to compare the osmolarity and to associate the function of the tear film in people with metabolic syndrome with those of a group without metabolic syndrome, unlike in our study, which investigated the associations between lipid levels within normal limits and tear breakdown time and aqueous secretion [15].

The control group, from Erdur et al., does not describe BMI since it follows the parameters defined by the International Diabetes Federation that establish the criterion to define nonmetabolic syndrome: waist circumference <80 cm in women and <90 cm in men. Surprisingly, for individuals postbariatric surgery, the same criteria defined by the International Diabetes Federation for identifying metabolic syndrome may not be applicable, especially those related to waist circumference, considering that one of the subsequent benefits reported in those who undergo bariatric surgery is lower lipid levels. For the above reasons, comparisons of blood components between groups have been made to determine possible data trends. Importantly, a larger waist circumference is associated with a higher BMI [20]. On the basis of the above, it can be estimated that the BMI of the control group described by Erdur et al. and the estimate made by Gierach et al. [21] should be at least 25.8 kg/m^2^.

Despite the above findings, since neither the Schirmer values between groups nor the HDL and triglyceride values differed considerably, the correlations likely showed the same trend: a lower Schirmer value was associated with a higher triglyceride level; therefore, water secretion from the lacrimal gland was lower. In addition, with respect to the BUT, there was a significant difference between the groups, with the value being lower within the gastric bypass surgery group, which could confirm the hypothesis that postgastric bypass surgery tear deficiencies could be mainly of evaporative origin and not watery. Importantly, both studies included participants with normal lipid values, but the inclusion criteria for the samples differed between the two studies, which could generate another source of variation. Additionally, Serrano Morales et al. reported that elevated total cholesterol and triglyceride values indicate a greater presence of dry eye symptoms, suggesting that cholesterol influences the pathogenesis of the meibomian gland [10]. Our results are consistent with those of the previous study, as total cholesterol and triglyceride levels, even within normal values, are inversely associated with the Schirmer test, which reflects the ability of the lacrimal gland to produce tears. Thus, increased levels of cholesterol and triglycerides seem to affect the production of the aqueous component of the tear.

According to the Dry Eye World Study 2017, the BUT values described in Table 1 for the group in this study presented values below normal; the same table describes the Schirmer values that are within normal values [8]. Therefore, dry eye in these participants may be of the evaporative type rather than the aqueous type, which could be due to meibomian gland dysfunction. Thus, high levels of cholesterol and triglycerides could be associated with aqueous-type dry eye (main gland production), and low HDL levels could be associated with evaporative-type dry eye (meibomian gland production). On the other hand, since the Schirmer values are negatively associated with cholesterol and triglyceride levels, an increase in these blood lipid values, in addition to being a factor in cardiometabolic disease, could affect the aqueous component of the tear film. Thus, maintaining triglyceride, cholesterol and high-density lipoprotein (HDL) levels within the values indicated as normal is important not only to reduce the risk of heart attack but also to promote improved and smooth movement of the ocular surface. Patients who undergo gastric bypass surgery may have a high likelihood of suffering dry eye or a modified lipid tear layer; however, whether dyslipidemia is responsible for developing DES remains unclear and highlights important topics to investigate further.

Furthermore, for routine control, patients who undergo gastric bypass surgery should be periodically evaluated by vision specialists or ophthalmologists to supervise tear film characteristics with the purpose of identifying dry eye manifestations that could be linked to dry eye syndrome [22,23]. This can significantly affect the quality of life of patients because several complications can lead to blindness. Some mechanisms involved in tear film production remain unknown, especially for patients who undergo gastric bypass surgery, as the results suggest that HDL levels above 40 seem to favor the secretion of the fatty layer of the tear film. These findings could be related to meibomian gland production. However, cholesterol and triglyceride levels, even within normal limits, appear to affect aqueous components, which are produced mainly by the main lacrimal gland. This opens the way for further research into ocular physiology and biochemistry in both postbariatric and nonbariatric patients. This is because even with normal lipid levels, meibomian gland dysfunction may be present.

## Figures and Tables

**Table 1:** Patient characteristics (*n* = 30).

Variables	Age	Weight	Height	BMI	Qx Time
Units	Years	kg	M	kg/m^2^	Years
Mean/SD	48.5 ± 9	81 ± 16	1.6 ± 0.1	32.7 ± 5.5	7.4 ± 2
Range	33–65	51.5–114.7	1.4–1.7	23.8–48.7	2.5–12

Control group Age 47 ± 7 (32 to 58 years) from Erdur et al., 2017 [15]. *T* Student’s test for age between groups *p* = 0.43, not statistically significant.

**Table 2:** Descriptive measurements of the tear film and blood levels.

		Gastric Bypass Surgery		Control **	Welch’s *T*-Test
Test	*n*	Mean/SD	Median	Mean/SD	*p* Value
BUT	30	5 ± 1.5	4.8	18.1 ± 0.5	<0.001
Schirmer *	30	16.6 ± 9.4	14.5	16.8 ± 2.6	0.91
HDL *	28	59.8 ± 16.5	58	54.3 ± 4.3	0.094
Triglycerides	27	93.3 ± 30	89	92 ± 6.8	0.826
Glucose *	30	89.4 ± 7.7	87	83 ± 3.6	
Cholesterol	29	154 ± 36	147		

** Control group from Erdur et al., 2017 [15]. * Does not meet the Shapiro–Wilk criteria for normality.

## Data Availability

The data presented in this study are available on request from the corresponding author.
